# Associations Between Ambient PM_2.5_ Levels and Children’s Pneumonia and Asthma During the COVID-19 Pandemic in Greater Jakarta (*Jabodetabek*)

**DOI:** 10.5334/aogh.4623

**Published:** 2025-02-24

**Authors:** Budi Haryanto, Bin Jalaludin, Al Asyary, Nathaniel Roestandy, Fajar Nugraha

**Affiliations:** 1 Department of Environmental Health, Faculty of Public Health, Universitas Indonesia, Indonesia; 2 Research Center for Climate Change, I-SER, Universitas Indonesia, Indonesia; 3 School of Population Health, University of New South Wales, Australia; 4 PT Nafas Aplikasi Indonesia, Indonesia; 5 Department of Biostatistics and Population, Faculty of Public Health, Universitas Indonesia, Indonesia

**Keywords:** children’s respiratory health, PM_2.5_ exposure, Greater Jakarta, asthma and pneumonia in children, air pollution and public health

## Abstract

*Background:* Children in Indonesia are especially vulnerable to air pollution due to their developing respiratory systems and unique exposure patterns. As one of the top 50 nations most at risk from environmental degradation, Indonesia faces significant public health concerns, especially in rapidly urbanizing areas such as Greater Jakarta, where emissions from transportation contribute heavily to pollution. This study investigates the relationship between PM_2.5_ exposure and cases of asthma and pneumonia in children across Greater Jakarta’s 11 cities and districts from 2020 to 2022, aiming to provide essential data for health planning and policy.

*Methods:* The data were collected from NafasID’s PM_2.5_ monitoring network and local health offices reporting monthly cases of asthma and pneumonia in children. Analytical methods included correlation and regression modeling to assess the association between air pollution and respiratory health across different regions. The results reveal a high number of respiratory disease, with 73,694 pneumonia and 15,825 asthma cases reported.

*Results:* Average PM_2.5_ concentration in Greater Jakarta was 42.5 µg/m^3^, with notable variation between areas. Bekasi District recorded the highest levels, while North Jakarta was lower. Depok City showed the strongest correlation between PM_2.5_ and pneumonia (*r* = 0.61, *p* = 0.004), indicating a sharp increase in cases with rising PM_2.5_, while other areas showed weaker correlations. Asthma cases had weak-to-moderate correlations with PM_2.5_, which is largely nonsignificant, suggesting complex factors beyond outdoor air pollution may influence asthma.

*Conclusion:* The findings highlight the critical need for improved air quality measures and targeted public health interventions. Addressing air pollution will be vital for reducing respiratory illness and supporting child health resilience in Indonesia’s urban centers.

## Background

Children are especially vulnerable to air pollutants compared with adults due to several differences that can lead to higher exposure to air pollution and higher doses of the pollutants reaching the lungs, such as the ongoing process of lung growth and development, incomplete metabolic systems, immature host defenses, high rates of infection by respiratory pathogens, and activity patterns specific to children [1]. In addition, the efficiency of detoxification systems exhibits a time‑dependent pattern during prenatal and postnatal lung development that in part accounts for the increased susceptibility of young children to pollutants at critical points in time [2–5]. The report finds that Indonesia is among the top 50 countries in the world where children are most at risk from climate change and environmental degradation [6]. Indonesian children are highly exposed to vector‑borne diseases, air pollution, and coastal floods, and also, investments in social services, particularly health and nutrition, education, and social protection and financial inclusion can make a significant difference in our ability to safeguard their futures from the impact of climate change. In Indonesia, the fourth most populated country in the world, children are under threat of exposure to contaminated water, air, food, and soil, which can cause gastrointestinal and respiratory diseases, birth defects, and neurodevelopmental disorders [6].

Rapid economic development in Indonesia has created severe air pollution problems, particularly in its big cities. Jakarta is known as the most polluted mega‑city after Mexico City and Bangkok. Its major source of air pollution is emissions from transportation [7, 8]. Diseases related to vehicular emissions and air pollution include acute respiratory infection, bronchial asthma, bronchitis, and eye and skin irritations and constituted 63% of all visits to health care centers [9–11]. It is believed that the lack of epidemiological evidence, particularly the effects of air pollution on children’s health, may lead to the lack of awareness of decision makers to the adverse effects of air pollution and, hence, the lack of appropriate strategies to protect the Greater Jakarta population from the exposure of air pollution [12].

Several studies on particulate matter less than or equal to a 2.5 µm in diameter (PM_2.5_) concentration in Jakarta revealed a noticeable decrease during the coronavirus disease 2019 (COVID‑19) pandemic. This decline is attributed to the implementation of large‑scale social restrictions (PSBB), which restricted outdoor activities. As a result, the number of vehicles on the streets sharply decreased, leading to improved air quality. The reduction in traffic emissions and seasonal variation played a significant role in lowering PM_2.5_ levels, highlighting the impact of reduced human activity on urban air pollution. [13, 14].

To strengthen and focus local government programs aimed at preventing, promoting, and controlling air pollution‑related diseases among children, there is an urgent need for an ecological study to assess the risks of such diseases across cities and districts. This study investigates the effects of ambient air pollution on children’s health, specifically examining the correlation between PM_2.5_ levels and asthma and pneumonia in 11 cities and districts of Greater Jakarta during the COVID‑19 pandemic, from March 2020 to December 2022.

## Method

An ecological study design was conducted by analyzing secondary data PM_2.5_ from NafasID and surveillance data on asthma and pneumonia in children from various cities/districts’ health offices. Both types of data were aggregated and compared monthly across 11 cities/districts in Greater Jakarta (Jabodetabek). Greater Jakarta, which includes North Jakarta, Central Jakarta, West Jakarta, East Jakarta, and South Jakarta, as well as the cities of Bogor, Depok, Tangerang, South Tangerang, and Bekasi and the districts of Bogor and Bekasi, is among the areas most severely affected by air pollution.

Daily mean PM_2.5_ concentration data were obtained from 105 monitoring sensors in Greater Jakarta operated by NafasID, using Airly sensors designed for outdoor conditions and calibrated for high accuracy with local reference stations [15]. NafasID manages Jabodetabek’s largest air quality sensor network. For each city/district, where four to seven monitoring sensors operated, the daily mean PM_2.5_ concentration data were used to calculate monthly means for further analysis. Monthly number of cases of asthma and pneumonia in children, collected by the Greater Jakarta’s health offices during the COVID‑19 pandemic (March 2020 to December 2022), were used for analysis. The data of asthma and pneumonia cases are based on the ICD‑10 report, which for asthma and pneumonia are J‑45 and J‑18.9, respectively. This study involved children’s pneumonia (73,694) cases and asthma (15,825). Statistical correlation and linear regression models were applied to estimate risks of asthma and pneumonia among children in the Greater Jakarta and in each city/district. We did not adjust for season, temperature, humidity, and precipitation.

## Results

Greater Jakarta, known as Jabodetabek, encompasses Jakarta and its surrounding 11 cities and districts. It is Indonesia’s largest metropolitan area and a bustling hub of economic, political, and cultural activities. With a population exceeding 30 million, it is one of the world’s most densely populated regions. The area is characterized by stark contrasts: modern skyscrapers and sprawling malls juxtaposed with informal settlements. Rapid urbanization has not only fueled economic growth but also created challenges, including traffic congestion, housing shortages, and environmental issues. Despite these hurdles, Greater Jakarta remains a vibrant and dynamic region, vital to Indonesia’s development and global presence.

**Figure 1:**
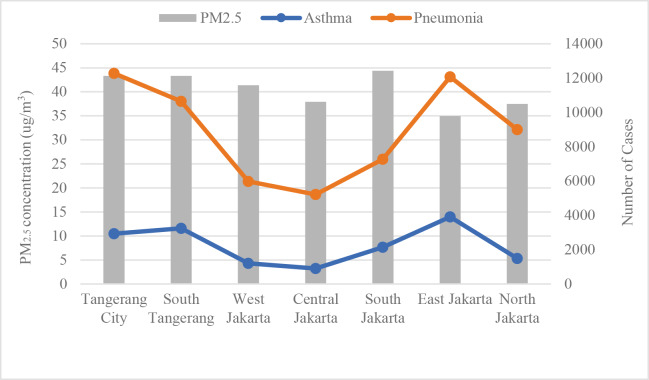
Annual average of PM_2.5_ concentration, children’s asthma and pneumonia cases in Greater Jakarta, 2020–2022.

Figure 1 shows the average PM_2.5_ concentration and reported asthma and pneumonia cases across Greater Jakarta from 2020 to 2022. Pneumonia cases (orange line) show a strong correlation with PM_2.5_ levels, peaking in Tangerang City and East Jakarta, where pollution is highest. Asthma cases (blue line) remain relatively stable across regions, with a slight increase in East Jakarta, indicating a potential sensitivity to higher pollution levels. Central Jakarta has lower asthma and pneumonia numbers despite moderate PM_2.5_ levels. The data suggest that regions with higher pollution experience more respiratory health issues, particularly pneumonia, highlighting the need for air quality management. The stable PM_2.5_ levels suggest that COVID‑19 interventions, rather than air pollution, significantly influenced pneumonia trends during this period.

During the COVID‑19 pandemic in Indonesia, from March 2020 to December 2022, the annual average PM_2.5_ concentration in Greater Jakarta was measured at 42.5 µg/m^3^, indicating a moderate air quality concern. Among cities and districts, North Jakarta recorded the lowest concentration at 35 µg/m^3^, suggesting relatively cleaner air compared with the region’s average. In contrast, Bekasi District had the highest PM_2.5_ levels, reaching 54.3 µg/m^3^, marking it as an area of heightened pollution. This variation highlights the uneven distribution of air quality across Greater Jakarta, with some districts experiencing significantly higher fine particulate matter concentrations, potentially affecting public health differently throughout the metropolitan area.

In Greater Jakarta’s 11 cities and districts, from 2020 to 2022, there were 73,694 cases of children’s pneumonia (incidence rate (IR) of 1.1%) and 15,825 cases of children’s asthma (IR of 0.5%), reported by city and district health offices. These high numbers highlight the urgent need for cleaner air initiatives to protect children’s health and reduce the burden of respiratory diseases in the region.

**Figure 2:**
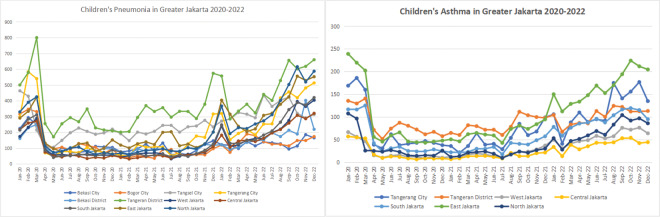
Monthly trend of children’s pneumonia and asthma during the COVID‑19 pandemic in Greater Jakarta, March 2020 to December 2022.

From 2020 to 2022, children’s pneumonia cases in Greater Jakarta varied significantly across regions, with peaks in early 2020 and late 2022. While cases initially decreased after mid‑2020, they gradually rose again, notably in the Bekasi District, Tangerang City, and Bogor City, indicating ongoing health challenges related to respiratory infections. Children’s asthma cases show a decline in early 2020, followed by a steady increase through late 2022. Tangerang City and North Jakarta experienced the highest rates, with notable peaks. This trend suggests ongoing respiratory health risks, likely influenced by environmental factors and air quality (Figure 2).

In Greater Jakarta, a rise of 15 µg/m^3^ in PM_2.5_ levels has been linked to a 4% increase in pneumonia cases (277 additional cases) and a 36% increase in asthma cases (75 additional cases). Among cities/districts, the regression model of PM_2.5_ and pneumonia incidence vary across regions. Depok City shows the strongest correlation (*R*^2^ = 0.37, *p* = 0.004), with the regression equation indicating a sharp increase in pneumonia cases with rising PM_2.5_ levels, in which an increasing of 15 µg/m^3^ PM_2.5_ was associated with 6.1% rise of pneumonia cases. Bekasi City also has a notable regression model (*R*^2^ = 0.29, *p* = 0.01) but with a weaker positive relationship. Central and South Jakarta exhibit moderate associations (*R*^2^ = 0.11 and 0.08, *p* = 0.02 and 0.04, respectively), showing a slight increase in pneumonia cases as PM_2.5_ rises. In contrast, cities such as Bogor and North Jakarta show weaker, statistically insignificant relationships, indicating less association between PM_2.5_ and pneumonia there (Table 1).

**Table 1:** Pearson correlation and regression analysis of PM_2.5_ with pneumonia and asthma by cities/districts in Greater Jakarta.

CITY/DISTRICT	OUTCOME	*R* ^2^	NUMBER AND PERCENT OF CASES PER 15 µG/M^3^ INCREASE IN PM_2.5_		*P*‑VALUE
			NO. OF CASES	%	
Bekasi City	Pneumonia	0.29	139	2.0	0.01
Bekasi District	Pneumonia	0.09	211	3.0	0.10
Tangerang City	Pneumonia	0.08	274	4.0	0.13
	Asthma	0.07	99	47.5	0.17
South Tangerang City	Pneumonia	0.04	289	4.2	0.22
Bogor City	Pneumonia	0.01	78	1.1	0.64
Depok City	Pneumonia	0.37	420	6.1	0.004
West Jakarta	Pneumonia	0.03	275	4.0	0.26
	Asthma	0.01	56	26.5	0.5
Central Jakarta	Pneumonia	0.11	245	3.5	0.02
	Asthma	0.07	47	22.7	0.06
South Jakarta	Pneumonia	0.08	305	4.4	0.04
	Asthma	0.06	102	49.0	0.09
East Jakarta	Pneumonia	0.07	361	5.2	0.07
	Asthma	0.03	174	83.1	0.21
North Jakarta	Pneumonia	0.02	395	5.7	0.39
	Asthma	0.01	72	34.5	0.64
Greater Jakarta	Pneumonia	0.02	277	4.0	0.002
	Asthma	0.001	75	36,0	0.91

The relationship between PM_2.5_ concentration and asthma rates in Greater Jakarta reveals weak‑to‑moderate correlations across different cities, though mostly statistically nonsignificant. Tangerang City has a weak correlation (*r* = 0.256, *p* = 0.17), with a regression suggesting a slight increase in asthma rates as PM_2.5_ rises, though the relationship is not statistically significant. Central Jakarta shows a similar pattern with *r* = 0.259 and a *p*‑value close to significance (*p* = 0.06), indicating a minor inverse association. South Jakarta has a weak correlation (*r* = 0.235, *p* = 0.09) with a slight negative relationship. West and North Jakarta, however, show very weak and statistically nonsignificant correlations (*r* = 0.111 and 0.07), implying minimal association between PM_2.5_ levels and asthma in those areas. Overall, PM_2.5_ may have some influence on asthma rates in certain districts, but the effect is generally weak.

## Discussion

During the COVID‑19 pandemic in Indonesia (from March 2020 to December 2022), the Greater Jakarta region reported 73,694 cases of children’s pneumonia and 15,825 cases of children’s asthma, a significant burden on public health. These data underscore the urgent need for targeted interventions to improve air quality and mitigate respiratory risks for children, who are particularly vulnerable to air pollution. Variations in disease patterns across different cities and districts within Greater Jakarta point to the complex interactions between pollution, local factors, and public health outcomes.

Children’s pneumonia cases during this period fluctuated across regions, with notable peaks in early 2020 and late 2022. Although cases declined after mid‑2020, the numbers rose again in Bekasi District, Tangerang City, and Bogor City, signaling persistent respiratory health challenges. The finding is confirmed by an umbrella review, which evaluated 33 systematic reviews on air pollution’s effects on children’s health, covering outcomes such as respiratory diseases, hypertension, and mortality, which found that exposure to air pollution significantly increases children’s risk of developing conditions such as leukemia, autism spectrum disorders, asthma, obesity, and eczema [15, 16]. A similar finding is also supported by a study in Taiwan (2024), which examined PM_2.5_ exposure’s effects on 4,736 children in central Taiwan and showed significant links to asthma, exercise‑induced wheezing, itchy eyes, and nasal issues. A 1 µg/m^3^ increase in PM_2.5_ was associated with a 1.0% rise in fractional exhaled nitric oxide (FENO), indicating increased airway inflammation [17]. Such patterns indicate that, while progress may have been made during parts of the pandemic, local environmental and perhaps socioeconomic factors continue to influence children’s pneumonia. The review underscores the need for strengthened child health care and pollution reduction efforts to improve intergenerational health equity.

Children’s asthma cases also showed a decline in early 2020 but gradually increased through late 2022, with Tangerang City and North Jakarta experiencing the highest rates and notable peaks. This trend suggests that the influence of PM_2.5_ on respiratory health may be complex and that asthma might be exacerbated by a combination of environmental and indoor air quality issues, potentially affected by household pollutants or other local sources.

The average PM_2.5_ concentration across Greater Jakarta from 2020 to 2022 was 42.5 µg/m^3^, reflecting a moderate air pollution level. However, significant disparities in PM_2.5_ levels exist between regions, with North Jakarta at the lower end (35 µg/m^3^) and Bekasi District at the higher end (54.3 µg/m^3^). This uneven distribution suggests that children’s exposure to air pollution—and, thus, their respiratory health risk—may differ significantly based on location. Bekasi’s higher PM_2.5_ levels correlate with its elevated pneumonia cases, suggesting a possible link between high pollution and respiratory infection risks in this district.

Examining the association between PM_2.5_ levels and children’s pneumonia across different areas reveals interesting trends [18]. Depok City exhibited the strongest correlation between PM_2.5_ and pneumonia, suggesting that even minor increases in PM_2.5_ concentration significantly impact pneumonia cases. In Bekasi City, a moderate association was observed, with regression analysis indicating a smaller increase in pneumonia as PM_2.5_ levels rise. South and Central Jakarta showed similar moderate correlations, although the relationship is somewhat weaker than in Depok and Bekasi. In contrast, cities such as Bogor and North Jakarta show much weaker associations, suggesting that air quality may play a smaller role in children’s pneumonia cases in these areas.

For children’s asthma, the relationship with PM_2.5_ appears weaker and less consistent across regions. Tangerang City has a weak correlation (*r* = 0.256, *p* = 0.17) but with an inverse association, suggesting slightly lower asthma rates as PM_2.5_ rises, which may imply influences from other factors beyond outdoor air pollution. Central Jakarta displays a similar trend, with an inverse correlation (*r* = 0.259, *p* = 0.06), while South Jakarta has a weak correlation (*r* = 0.235, *p* = 0.09) with a slight decrease in asthma rates associated with higher PM_2.5_. Meanwhile, West and North Jakarta show minimal associations, indicating that in these areas, asthma rates may be less impacted by PM_2.5_ levels. This finding is similar with the findings of a systematic review by the Global Burden of Disease [19] and Allun et al. [20], which suggest a negative correlation between short‑term PM_2.5_ exposure and a range of lung function indicators in children diagnosed with asthma [14, 21].

## Conclusion

The data show a more consistent link between PM_2.5_ and pneumonia compared with asthma, with the strongest associations in cities with higher pollution. These findings underscore the need for more granular research into other factors contributing to respiratory illnesses, as well as the importance of localized air quality interventions to protect children’s health across Greater Jakarta (Table 2).

**Table 2:** Recommendations to address public health concern in Greater Jakarta.

INTERVENTIONS	IMPLEMENTATIONS AND IMPLICATIONS
Strengthen Air Quality Monitoring and Data Granularity	Enhanced air quality monitoring at a neighborhood level to identify pollution hotspots. Expanding the number of real‑time PM_2.5_ sensors and integrating pollution data with health records can help researchers and policymakers better understand localized impacts.
Implement Targeted Pollution Control Measures	Stricter emissions regulations should be enforced, particularly for industrial zones and high‑traffic areas. This includes phasing out high‑emission vehicles, enforcing low‑emission zones, and strengthening regulations on industrial pollutants.
Develop Community‑Specific Health Interventions	Localized interventions, such as distributing air purifiers to households in high‑risk areas and promoting the use of protective masks during high‑pollution periods, can help mitigate health risks. Schools and childcare centers should also be prioritized for improved indoor air filtration systems.
Expand Public Awareness Campaigns	Educational initiatives should inform parents and caregivers about the dangers of PM_2.5_ exposure and effective preventive measures. Public health campaigns can encourage behavioral changes, such as limiting outdoor activities during high‑pollution days and adopting cleaner cooking practices.
Invest in Long‑Term Research on Respiratory Health	Further research is needed to explore additional environmental and socioeconomic factors contributing to pneumonia and asthma. Longitudinal studies can provide deeper insights into the long‑term health impacts of PM_2.5_ exposure and guide evidence‑based policy decisions.
